# An Overview of FGF-23 as a Novel Candidate Biomarker of Cardiovascular Risk

**DOI:** 10.3389/fphys.2021.632260

**Published:** 2021-03-09

**Authors:** Sara Vázquez-Sánchez, Jonay Poveda, José Alberto Navarro-García, Laura González-Lafuente, Elena Rodríguez-Sánchez, Luis M. Ruilope, Gema Ruiz-Hurtado

**Affiliations:** ^1^Cardiorenal Translational Laboratory, Institute of Research i+12, Hospital Universitario 12 de Octubre, Madrid, Spain; ^2^CIBER-CV, Hospital Universitario 12 de Octubre, Madrid, Spain; ^3^School of Doctoral Studies and Research, European University of Madrid, Madrid, Spain

**Keywords:** FGF-23, FGFR, heart, LVH, heart failure – pharmacological treatment – systolic dysfunction

## Abstract

Fibroblast growth factor-23 (FGF)-23 is a phosphaturic hormone involved in mineral bone metabolism that helps control phosphate homeostasis and reduces 1,25-dihydroxyvitamin D synthesis. Recent data have highlighted the relevant direct FGF-23 effects on the myocardium, and high plasma levels of FGF-23 have been associated with adverse cardiovascular outcomes in humans, such as heart failure and arrhythmias. Therefore, FGF-23 has emerged as a novel biomarker of cardiovascular risk in the last decade. Indeed, experimental data suggest FGF-23 as a direct mediator of cardiac hypertrophy development, cardiac fibrosis and cardiac dysfunction via specific myocardial FGF receptor (FGFR) activation. Therefore, the FGF-23/FGFR pathway might be a suitable therapeutic target for reducing the deleterious effects of FGF-23 on the cardiovascular system. More research is needed to fully understand the intracellular FGF-23-dependent mechanisms, clarify the downstream pathways and identify which could be the most appropriate targets for better therapeutic intervention. This review updates the current knowledge on both clinical and experimental studies and highlights the evidence linking FGF-23 to cardiovascular events. The aim of this review is to establish the specific role of FGF-23 in the heart, its detrimental effects on cardiac tissue and the possible new therapeutic opportunities to block these effects.

## Introduction

Cardiovascular disease remains the leading cause of morbidity and mortality worldwide, and heart failure (HF) has become a major public health problem in the industrialised world (Members et al., 2012). The prevalence of coronary artery disease in the general population is 7.3% (Enbergs et al., 2000), while it is around 15–20% for left ventricular hypertrophy (LVH) (Weber, 1991) and 3.9% for HF (Mosterd et al., 1999). Recent research provides evidence that a high plasma level of fibroblast growth factor-23 (FGF)-23 is a hallmark of cardiac damage resulting in deleterious remodelling and the induction of cardiac hypertrophy. In the last few years, FGF-23 has been identified as a new trigger of cardiac dysfunction (Seiler et al., 2011; Leifheit-Nestler et al., 2018b; Navarro-García et al., 2019). Therefore, it is essential to not only understand the mechanisms whereby FGF-23 signals are transduced but also the variables associated with the increase in FGF-23 so new therapeutic strategies can be developed and applied in the near future (Chonchol et al., 2016; Rodelo-Haad et al., 2018).

FGF-23 is a hormone mainly synthesised by osteocytes and osteoblasts in long bones, although it can also be produced and secreted by different tissues, such as liver or even heart tissue under stress conditions (Hu et al., 2013). FGF-23 is involved in phosphate homeostasis and promotes renal phosphate excretion (Wolf, 2012; Mace et al., 2015), downregulation of 1,25-dihydroxyvitamin D (vitamin D) synthesis (Krajisnik et al., 2007; Mace et al., 2015; Navarro-García et al., 2018) and a decrease in parathyroid hormone (PTH) secretion (Navarro-García et al., 2018; von Jeinsen et al., 2019). FGF-23 is a 32 kDa protein encoded by the gene *fgf23* located in chromosome 12 and belongs to the FGF subfamily FGF19, which corresponds to the endocrine FGFs (Hu et al., 2013). It appears in the circulating blood in two forms: intact full-length proteins and C-terminal proteins resulting from the rupture of the entire molecule by furin-like enzymes (Tagliabracci et al., 2014). FGF receptors (FGFRs) mediate the effects of FGF-23 and have tyrosine-kinase activity (Farrell and Breeze, 2018). There are four isoforms (FGFR1–4), but FGF-23 has a low affinity for all FGFRs and requires a cofactor to promote its union (Kurosu and Kuro-o, 2009). FGFRs are widely expressed in various tissues, including bone, kidney and heart tissues. In the kidneys, FGF-23 binds to FGFR1 using protein Klotho as a cofactor (Kurosu and Kuro-o, 2009); however, in the heart, FGF-23 binds to FGFR4 independently of Klotho (Grabner et al., 2015; Faul, 2017). Therefore, whether FGF-23 exerts its effect on the heart through an unknown cofactor or it does not need the cofactor link remains unclear (Urakawa et al., 2006).

FGF-23 was originally identified through a gene mutation in patients with autosomal dominant hypophosphatemic rickets or X-linked hypophosphatemic rickets that results in elevated serum levels of FGF-23 (White et al., 2000). The discovery of FGF-23 has revolutionised our understanding of mineral metabolism. Multilevel feedback and the kinetics of hormone actions within the bone–kidney–parathyroid loops system allow the controlled expression or action of the mineral–bone components, such as phosphates, PTH and vitamin D (Blau and Collins, 2015). Changes in any of these components lead to a misbalance in the feedback loops, altering the circulating levels of all these components and affecting several organs, including off-target organs such as the heart (Navarro-García et al., 2018). For instance, in chronic kidney disease (CKD), as kidney excretion capacity decreases, serum phosphate levels increase and stimulate FGF-23 synthesis, causing a rise of PTH and a deficiency of vitamin D, which can lead to adverse cardiovascular events like LVH and HF (Faul et al., 2011; Mathew et al., 2014; Navarro-García et al., 2018).

Recent evidence from human and experimental studies points to a key role of FGF-23 in the development and progression of cardiac disease. Significant changes in FGF-23 secretion and its action on the heart may have broad health implications that need to be elucidated. This review summarises our current understanding of FGF-23 and focuses on emerging areas of FGF-23 in human cardiac events and its intracellular pathways in cardiomyocytes and fibroblasts.

## Clinical Variables and Cardiac Events Associated With High Circulating FGF-23 Levels

Systemic levels of FGF-23 are commonly analysed as an important biomarker for the diagnosis and prognosis of mineral bone disorders (MBD), and its measurement is recommended for patients with CKD. Currently, the extra-renal effects of this protein are becoming even more relevant, finding other targets such as the parathyroid gland and the cardiovascular and immune systems (Haffner and Leifheit-Nestler, 2017). The role of FGF-23 in cardiac disturbances is not clear or defined, especially the effects related not only to structural changes in the myocardium but also those related to cardiac dysfunction. High circulating levels of FGF-23 have been associated with cardiovascular pathophysiologic events, such as LVH, endothelial dysfunction and atherosclerosis (Lutsey et al., 2014). In addition, it remains unknown whether FGF-23 plays a detrimental role in cardiovascular events or if high plasma levels of FGF-23 are a simple consequence of cardiac disturbances. In this review, we identify the agreements and contradictions between clinical studies and put forward a scenario in which the specific role of FGF-23 in cardiovascular diseases can also be significantly harmful. Moreover, another unknown aspect that has arisen is the variability in FGF-23 levels according to different demographic variables, such as age, gender, ethnicity and clinical background. This issue is described in the following section.

### FGF-23 and Demographic Variables: Age, Sex, and Ethnicity

High plasma levels of FGF-23 are associated with older age groups (>60 years) (Ix et al., 2012; Kestenbaum et al., 2014; Lutsey et al., 2014; Wright et al., 2014; Masson et al., 2015; Panwar et al., 2015; Speer et al., 2015; Souma et al., 2016; Ter Maaten et al., 2018; Huo et al., 2019), although some studies in younger populations have not found a significant correlation with age (Parker et al., 2010; Agarwal et al., 2014; di Giuseppe et al., 2014; Poelzl et al., 2014; Di Giuseppe et al., 2015; Chonchol et al., 2016; Takahashi et al., 2018). As age increases, metabolic disturbances are more prevalent; therefore, the lack of correlation with age may be due to the lack of metabolic changes. Another reason could be the low Klotho levels observed in older populations. Klotho is an anti-ageing factor, mainly expressed in the kidneys, which functions as a coreceptor of FGF-23 to increase the renal excretion of phosphates (Bian et al., 2014). As Klotho expression declines, serum phosphates increase and stimulate bone FGF-23 production, e.g., in CKD patients (Drew et al., 2017).

Interestingly, although the cardiovascular risk is higher in men, elevated FGF-23 levels are more commonly correlated with women (Parker et al., 2010; Seiler et al., 2011; Ix et al., 2012; Agarwal et al., 2014; di Giuseppe et al., 2014; Wright et al., 2014; Di Giuseppe et al., 2015; Masson et al., 2015; Panwar et al., 2015; Speer et al., 2015; Souma et al., 2016; Reindl et al., 2017; Ter Maaten et al., 2018; Cheng et al., 2020) than men (Kestenbaum et al., 2014; Chonchol et al., 2016; Huo et al., 2019). Nevertheless, some authors suggest that levels of FGF-23 could be independent of sex (Shibata et al., 2013; Lutsey et al., 2014; Poelzl et al., 2014; Koller et al., 2015). This discordance could be explained by the fact that most studies were carried out with older patients, including post-menopausal women. It is known that oestrogens diminish plasma FGF-23 levels, which might explain the increased FGF-23 levels in post-menopausal women compared to men (Ix et al., 2011). The physiological reason for the oestrogen effect on FGF-23 levels is that the oestrogen receptor regulates urinary phosphorus excretion at the proximal tubule, so in post-menopausal women, serum phosphorus levels are increased, thereby increasing FGF-23 secretion (Ix et al., 2011).

In contrast, the relationship between ethnicity and high plasma levels of FGF-23 is not yet clear. Some studies have associated greater levels of FGF-23 with Caucasian ethnicity (Ix et al., 2012; Chonchol et al., 2016; Patel et al., 2020), others with African American or Hispanic ethnicities (Lutsey et al., 2014; Wright et al., 2014), and others with none of them (Parker et al., 2010; Kestenbaum et al., 2014; Panwar et al., 2015; Robinson-Cohen et al., 2020). For instance, studies carried out in very diverse populations did not find any correlation with ethnicity variables (Kestenbaum et al., 2014). In this sense, Gutiérrez et al. (2010) demonstrated that a high level of serum phosphates, which leads to increased FGF-23 levels, is associated with low socioeconomic status independently of ethnicity. Some studies have even pointed to a relationship between FGF-23 levels and industrialisation, possibly due to different phosphate dietary intake through the addition of phosphates as food preservatives (Yuen et al., 2016). Therefore, future research and clinical studies should incorporate all these variables to achieve an intersectional perspective of the levels of FGF-23 in the general population (Table 1).

**TABLE 1:** Demographic and clinical variables correlated with high plasma levels of FGF-23.

Variable	Condition	References
**Demographic variables**		
Age	Older > 60 years	Lutsey et al., 2014
		Panwar et al., 2015
		Masson et al., 2015
		Ix et al., 2012
		Wright et al., 2014
		Kestenbaum et al., 2014
		Speer et al., 2015
		Souma et al., 2016
		Ter Maaten et al., 2018
		Huo et al., 2019
	Not Correlated	Di Giuseppe et al., 2015
		di Giuseppe et al., 2014
		Parker et al., 2010
		Poelzl et al., 2014
		Agarwal et al., 2014
		Chonchol et al., 2016
		Takahashi et al., 2018
Sex	Women	Di Giuseppe et al., 2015
		Panwar et al., 2015
		Masson et al., 2015
		di Giuseppe et al., 2014
		Ix et al., 2012
		Wright et al., 2014
		Parker et al., 2010
		Speer et al., 2015
		Souma et al., 2016
		Ter Maaten et al., 2018
		Seiler et al., 2011
		Reindl et al., 2017
		Agarwal et al., 2014
		Cheng et al., 2020
	Men	Kestenbaum et al., 2014
		Chonchol et al., 2016
		Huo et al., 2019
	Not Correlated	Koller et al., 2015
		Lutsey et al., 2014
		Shibata et al., 2013
		Poelzl et al., 2014
Ethnicity	Caucasian	Ix et al., 2012
		Chonchol et al., 2016
		Patel et al., 2020
	African American or Hispanic	Lutsey et al., 2014
		Wright et al., 2014
	Not Correlated	Panwar et al., 2015
		Robinson-Cohen et al., 2020
		Parker et al., 2010
		Kestenbaum et al., 2014

### FGF-23 and Adverse Cardiac Events

FGF-23 is an early and complementary predictor of adverse cardiac events and could be suitable for improving risk assessment in vulnerable patients with HF or reduced ejection fraction (Koller et al., 2015). FGF-23 is positively correlated with but does not directly depend on the classical biomarkers of cardiac damage, such as N-terminal-pro-B-type natriuretic peptide (NT-proBNP) (Seiler et al., 2011; Shibata et al., 2013; Kestenbaum et al., 2014; Poelzl et al., 2014; Koller et al., 2015; Masson et al., 2015; Panwar et al., 2015; Speer et al., 2015; Wohlfahrt et al., 2015; Ter Maaten et al., 2018; von Jeinsen et al., 2019; Song et al., 2021), high-sensitive cardiac troponin T (hs-cTnT) (Kestenbaum et al., 2014; Masson et al., 2015; Song et al., 2021) and C-reactive protein (CRP) (Parker et al., 2010; Seiler et al., 2011; Ix et al., 2012; Lutsey et al., 2014; Panwar et al., 2015; Speer et al., 2015; Reindl et al., 2017; Song et al., 2021). Furthermore, it has already been demonstrated that the predictive value of the combination of these biomarkers on cardiovascular risk assessment is significantly greater than any of them alone (Wohlfahrt et al., 2015). In specific clinical circumstances, however, several authors have noted the relevance of FGF-23 as an independent biomarker. In this sense, Speer et al. (2015) demonstrated that the predictive value of FGF-23 for mortality and complications after cardiac surgery is comparable and even greater than the European System for Cardiac Operative Risk Evaluation (EuroSCORE II) and as good as that of NT-proBNP.

Elevated systemic FGF-23 levels have been strongly associated with an increased risk of mortality, including cardiovascular mortality (Parker et al., 2010; Ix et al., 2012; Ärnlöv et al., 2013b; Poelzl et al., 2014; Masson et al., 2015; Speer et al., 2015; Chonchol et al., 2016; Ter Maaten et al., 2018; von Jeinsen et al., 2019). Indeed, several studies have shown that patients with high plasma levels of FGF-23 have a higher incidence of cardiovascular events and less survival (Parker et al., 2010; Masson et al., 2015; Speer et al., 2015). This fact could explain the increased mortality risk in these patients. In this sense, several authors have associated high plasma levels of FGF-23 with a greater risk of cardiovascular death than other causes of death (Ärnlöv et al., 2013a; Lutsey et al., 2014; Souma et al., 2016; Silva et al., 2019). Marthi et al. (2018) carried out a meta-analysis that looked at thirty-four studies: 17 with the general population, nine with CKD non-dialysis patients and eight with the dialysis population. Overall, when comparing participants classified by FGF-23 quartiles, in the top *versus* bottom third of baseline FGF-23 concentration there was a 70% increased risk of all-cause mortality and 42% increased risk of cardiovascular mortality (Marthi et al., 2018).

In general terms, the increased mortality risk associated with FGF-23 may be a consequence of the augmented incidence of cardiovascular events in patients with higher serum FGF-23 levels. LVH, HF, arrhythmias, myocardial infarction (MI) and vascular alterations such as stroke and vascular calcification are the most common adverse and fatal cardiac events studied in relation to FGF-23 (Table 2). It is important to highlight, however, that higher levels of FGF-23 have been associated not only with cardiovascular mortality but also with a significant increase in the risk of non-cardiovascular causes of death. Therefore, FGF-23 should be considered a relevant and specific predictor of mortality, and the relevance of FGF-23 as a predictor of mortality will most likely increase in the coming years.

**TABLE 2:** Risk of cardiac events and high plasma levels of FGF-23.

Adverse cardiac event	Association with	References
Mortality	Association with all-cause death	Masson et al., 2015
		Ix et al., 2012
		Parker et al., 2010
		Speer et al., 2015
		Chonchol et al., 2016
		Ter Maaten et al., 2018
		Ärnlöv et al., 2013b
		Poelzl et al., 2014
		von Jeinsen et al., 2019
	Association with cardiovascular death	Souma et al., 2016
		Ärnlöv et al., 2013a
		Lutsey et al., 2014
		Silva et al., 2019
LVH or LV mass	Association: Yes	Masson et al., 2015
		Silva et al., 2019
		Kestenbaum et al., 2014
		Shibata et al., 2013
		Agarwal et al., 2014
		Seiler et al., 2011
		Reindl et al., 2017
		Mirza et al., 2009
		Panwar et al., 2015
		Gutiérrez et al., 2008
		Patel et al., 2020
		Cheng et al., 2020
	Association: No	Wohlfahrt et al., 2015
Heart failure	Association: Yes	Lutsey et al., 2014
		Ix et al., 2012
		Parker et al., 2010
		Robinson-Cohen et al., 2020
		di Giuseppe et al., 2014
		Marthi et al., 2018
		Kestenbaum et al., 2014
		Andersen et al., 2016
		Ferreira et al., 2020
		Ferreira et al., 2019
		Cheng et al., 2020
	Association with LVEF < 40%	Seiler et al., 2011
		Shibata et al., 2013
		Poelzl et al., 2014
		von Jeinsen et al., 2019
		Agarwal et al., 2014
		Song et al., 2021
	Association with more severe NYHA class	Koller et al., 2015
		Wohlfahrt et al., 2015
		Ter Maaten et al., 2018
		Poelzl et al., 2014
Arrhythmias	Atrial fibrillation	Panwar et al., 2015
		Seiler et al., 2011
		Masson et al., 2015
		Ter Maaten et al., 2018
		Mathew et al., 2014
		Alonso et al., 2014
		Chua et al., 2019
		Dong et al., 2019
	Other arrhythmias	No evidence
Myocardial infarction	Association: Yes	Di Giuseppe et al., 2015
		Lutsey et al., 2014
		Ärnlöv et al., 2013b
		Marthi et al., 2018
		Takahashi et al., 2018
		Ferreira et al., 2020
	Association: No	Ix et al., 2012
		Parker et al., 2010
		Taylor et al., 2011
Stroke	Association: Yes	Di Giuseppe et al., 2015
		Panwar et al., 2015
		Wright et al., 2014
	Association: No	Kestenbaum et al., 2014
Vascular calcification	Association: Yes	Donate-Correa et al., 2019
		Silva et al., 2015
		Jimbo et al., 2014
		Dzgoeva et al., 2016
		Bortnick et al., 2019

*For more details see each article which contains hazard ratio (HR) of specific variables with 95% confidence interval (CI).*

#### FGF-23 and LVH

FGF-23 was associated with the development of LVH in CKD patients for the first time in 2009 by Gutiérrez et al. (2009), and this was corroborated by Faul et al. (2011). Currently, several studies have associated high plasma levels of FGF-23 with increased left ventricular mass (LVM), and consequently, with the degree of cardiac hypertrophy developed in the general population (Gutiérrez et al., 2008; Mirza et al., 2009; Seiler et al., 2011; Shibata et al., 2013; Agarwal et al., 2014; Kestenbaum et al., 2014; Masson et al., 2015; Panwar et al., 2015; Reindl et al., 2017; Silva et al., 2019; Cheng et al., 2020; Patel et al., 2020). In particular, FGF-23 has been linked to a greater risk of concentric hypertrophy, which is apparently a compensated cardiac hypertrophy that results in increased wall thickness without ventricular dilation (Mirza et al., 2009; Silva et al., 2019). In addition, it has been demonstrated that serum FGF-23 levels are associated with LVM and LVH in the older population (Mirza et al., 2009) and with less left ventricular ejection fraction and LVM in cardiology inpatients (Shibata et al., 2013). Therefore, the relationship between FGF-23 and cardiac hypertrophy, as well as the former’s direct effect on cardiac remodelling through molecular pathways, has been well established and is discussed in the experimental section (section 3.2).

#### FGF-23 and HF

It is well established that LVH causes pathologic changes in heart structure and function, increasing the risk of adverse cardiovascular events such as arrhythmias, coronary disease and HF. Indeed, elevations in circulating FGF-23 levels have been shown to have a strong relationship with HF (Parker et al., 2010; Kestenbaum et al., 2014; Lutsey et al., 2014; Ferreira et al., 2019, 2020; Cheng et al., 2020; Robinson-Cohen et al., 2020). HF is a clinical syndrome caused by a structural and/or functional cardiac abnormality and is characterised by the inability of the heart to supply the peripheral tissue with enough blood and oxygen (Atherton et al., 2016). It is one of the most common causes of morbidity and mortality (Ziaeian and Fonarow, 2016). Andersen et al. (2016) analysed serum FGF-23 in patients with HF *versus* healthy subjects and showed that FGF-23 levels were significantly higher in HF patients (1526 ± 1601 *versus* 55 ± 20 RU/mL, *p* = 0.007). Focusing on myocardial tissue, these authors also compared intracardiac FGF-23 expression levels between HF patients and healthy subjects but no differences were found (Andersen et al., 2016). These results suggest that increased plasma, but not cardiac FGF-23 levels might play an important role in the development of HF. However, as FGF-23 can be synthesised at the intracardiac level under pathological or stress circumstances such as MI, the contribution of cardiac FGF-23 should be further studied (Andrukhova et al., 2015).

In addition, Di Giuseppe et al. (2015) demonstrated that the relationship between FGF-23 levels and the development of HF was independent of other established risk factor markers, such as NT-proBNP. Ix et al. (2012) also showed that this relationship was stronger when kidney function was impaired, possibly due to the extremely high serum FGF-23 levels found in patients with kidney dysfunction (Wolf, 2010, 2012). Nevertheless, the meta-analysis by Marthi et al. (2018) did not find a trend across different levels of kidney function in the association between high FGF-23 and HF. From a cardiac functional point of view, many studies have correlated high plasma levels of FGF-23 with a reduced left ventricular ejected fraction (LVEF; <40%), which indicates that high FGF-23 levels are directly linked to systolic dysfunction (Seiler et al., 2011; Shibata et al., 2013; Agarwal et al., 2014; Poelzl et al., 2014; von Jeinsen et al., 2019; Song et al., 2021). Furthermore, high plasma levels of FGF-23 are also associated with albuminuria in CKD which is strongly associated with Heart Failure with reduced Ejection Fraction (HFrEF), but not with Heart Failure with preserved Ejection Fraction (HFpEF) (Nayor et al., 2017). However, several articles have shown that increased FGF-23 levels are associated with both types of HF, HFpEF (Almahmoud et al., 2018; van de Wouw et al., 2019; Kanagala et al., 2020; Roy et al., 2020) and HFrEF (Koller et al., 2015; Gruson et al., 2017). Thus, further studies are required to clarify the role of FGF-23 in each HF subtype. HF symptoms are graduated in the New York Heart Association (NYHA) functional classification based on the limitations to the physical activity of the patients caused by cardiac symptoms (Atherton et al., 2016). Importantly, elevations in FGF-23 levels have been associated with the most severe classes on the NYHA scale (Poelzl et al., 2014; Koller et al., 2015; Wohlfahrt et al., 2015; Ter Maaten et al., 2018; von Jeinsen et al., 2019) and, therefore, with more severe cardiac functional impairment and more serious stages of HF.

#### FGF-23 and Arrhythmia: Atrial Fibrillation

FGF-23 has been related to heart rate disturbances causing arrhythmias, which can be classified as extra beats, supraventricular tachycardia including atrial fibrillation (AF), ventricular arrhythmias and bradyarrhythmias. The vast majority of the clinical studies have correlated high plasma levels of FGF-23 with AF (Seiler et al., 2011; Alonso et al., 2014; Mathew et al., 2014; Masson et al., 2015; Panwar et al., 2015; Ter Maaten et al., 2018; Chua et al., 2019; Dong et al., 2019). According to Mathew et al. (2014), each two-fold increase in circulating FGF-23 increased the risk of AF by 41% in their multi-ethnic study of atherosclerosis and by 30% in their cardiovascular health study. There are several mechanisms by which high plasma levels of FGF-23 could cause AF. FGF-23 induces changes in the cardiac structure such as LVH and is also linked to vascular calcification (Mathew et al., 2014). Both factors modify filling pressures, thus increasing atrial size, the main risk factor for AF (Mathew et al., 2014). Evidence of other types of rhythm alterations, such as those related to ventricular arrhythmia, is yet to be provided in a clinical setting, however.

#### FGF-23 and MI

Myocardial infarction is associated with severe complications, such as HF, pericarditis, arrhythmias, ventricular aneurysm and septal defects (Fox et al., 1979). The relationship between high plasma levels of FGF-23 and MI is controversial and requires further research to conclude whether high plasma levels of FGF-23 increase MI risk or not. Some authors have found a significant increase in the levels of FGF-23 after an MI (Ärnlöv et al., 2013b; Lutsey et al., 2014; Di Giuseppe et al., 2015; Takahashi et al., 2018; Ferreira et al., 2019); however, other authors have described a weak increase (Marthi et al., 2018) and some did not find any variation in FGF-23 at all (Parker et al., 2010; Taylor et al., 2011; Ix et al., 2012). At present, circulating FGF-23 levels in patients who have suffered an MI are being studied in three clinical trials that will shed light on this issue and lead to important future conclusions (reported at www.clinicaltrials.gov: NCT01971619, NCT02548364, and NCT03405207).

#### FGF-23 and Stroke

FGF-23 not only has a harmful effect on the heart but also on the vasculature, as shown by its relation to strokes (Parker et al., 2010; Ärnlöv et al., 2013b; Wright et al., 2014; Di Giuseppe et al., 2015; Marthi et al., 2018). Although Kestenbaum et al. (2014) found a non-significant relationship between elevated FGF-23 and all-cause stroke, Wright et al. (2014) found that elevated FGF-23 was a risk factor independent of CKD for overall stroke in a racially and ethnically diverse urban community. It is important to note that there are different types of strokes depending on the triggering cause: ischemic strokes, which include thrombotic strokes and cardioembolic strokes; haemorrhagic strokes; transient ischemic attacks; and cryptogenic strokes. A relationship has only been demonstrated between FGF-23 and haemorrhagic stroke (Di Giuseppe et al., 2015) and FGF-23 and cardioembolic stroke (Panwar et al., 2015), however. Therefore, although stroke is beyond the scope of this review, the harmful effects of FGF-23 on the vasculature deserve to be mentioned.

#### FGF-23 and Vascular Calcification

Vascular calcification is a major risk factor for cardiovascular disease which contributes to the high incidence of cardiovascular mortality (Pescatore et al., 2019). FGF-23, as a mineral metabolism factor, plays a direct role in calcification of tissues by modulating disturbances in calcium and phosphate balance (Donate-Correa et al., 2019). The relationship between increased systemic FGF-23 levels and vascular calcification has been shown experimentally in *in vitro* approaches (Jimbo et al., 2014), in patients with established cardiovascular disease (Donate-Correa et al., 2019), in pre-dialysis diabetic (Silva et al., 2015) and in CKD patients (Dzgoeva et al., 2016). Hence, FGF-23 outstands as a promising prognostic biomarker and therapeutic target in vascular calcification.

Consequently, FGF-23 may be considered as a biomarker and predictor of several important cardiac adverse events, but the mechanisms by which FGF-23 could have a harmful effect directly or indirectly on the heart remain misunderstood.

### Other Disorders and Comorbidities Associated With High Plasma Levels of FGF-23

Finally, several clinical studies have linked the baseline characteristics of individuals with high plasma levels of FGF-23, discovering a direct correlation with bad health habits, MBD and CKD and with comorbidities such as diabetes and hypertension (Table 3).

**TABLE 3:** Other disorders and comorbidities correlated with high plasma levels of FGF-23.

**Variable**	**Condition**	**References**
**Lifestyle**		
Current smoker	Current smokers	Di Giuseppe et al., 2015
		Panwar et al., 2015
		Masson et al., 2015
		di Giuseppe et al., 2014
		Ix et al., 2012
		Parker et al., 2010
		Souma et al., 2016
		Seiler et al., 2011
		Agarwal et al., 2014
		Patel et al., 2020
	Not correlated	Kestenbaum et al., 2014
		Ärnlöv et al., 2013a
Physical activity	Worse	Masson et al., 2015
		Ix et al., 2012
		Kestenbaum et al., 2014
	Not correlated	Andersen et al., 2016
Inadequate food intake	Phosphate intake	Yuen et al., 2016
		Pool et al., 2020
**Metabolic bone disorders and kidney disease**		
Phosphate	Correlated positively	Di Giuseppe et al., 2015
		Lutsey et al., 2014
		Panwar et al., 2015
		Wright et al., 2014
		Parker et al., 2010
		Kestenbaum et al., 2014
		Poelzl et al., 2014
		Souma et al., 2016
		Seiler et al., 2011
		Shibata et al., 2013
		Taylor et al., 2011
		Reindl et al., 2017
		Agarwal et al., 2014
		Brandenburg et al., 2014
	Not correlated	Takahashi et al., 2018
Calcium	Correlated positively	Di Giuseppe et al., 2015
		Panwar et al., 2015
		Wright et al., 2014
		Parker et al., 2010
		Kestenbaum et al., 2014
		Reindl et al., 2017
		Patel et al., 2020
	Not correlated	Agarwal et al., 2014
		Takahashi et al., 2018
PTH	Correlated positively	Di Giuseppe et al., 2015
		Lutsey et al., 2014
		di Giuseppe et al., 2014
		Brandenburg et al., 2014
		Wright et al., 2014
		Souma et al., 2016
		Poelzl et al., 2014
		Taylor et al., 2011
		Sharma et al., 2020
	Not correlated	Shibata et al., 2013
Vitamin D	Correlated negatively	Souma et al., 2016
		Shibata et al., 2013
		Poelzl et al., 2014
		Chonchol et al., 2016
	Not correlated	Masson et al., 2015
		Kestenbaum et al., 2014
		Taylor et al., 2011
eGFR (<60 mL/min/1.73 m^2^)	Correlated negatively	Koller et al., 2015
		Lutsey et al., 2014
		Panwar et al., 2015
		Ix et al., 2012
		Wright et al., 2014
		Parker et al., 2010
		Kestenbaum et al., 2014
		Speer et al., 2015
		Souma et al., 2016
		Wohlfahrt et al., 2015
		Ter Maaten et al., 2018
		Seiler et al., 2011
		Shibata et al., 2013
		Ärnlöv et al., 2013b
		Agarwal et al., 2014
		Ärnlöv et al., 2013a
		Poelzl et al., 2014
		Brandenburg et al., 2014
		Sharma et al., 2020
		Patel et al., 2020
		von Jeinsen et al., 2019
	Not correlated	Di Giuseppe et al., 2015
		Andersen et al., 2016
Albumin-to-creatinine ratio or creatine levels	Correlated positively	Koller et al., 2015
		Panwar et al., 2015
		Masson et al., 2015
		Ix et al., 2012
		Wright et al., 2014
		Kestenbaum et al., 2014
		Speer et al., 2015
		Souma et al., 2016
		Shibata et al., 2013
		Taylor et al., 2011
		Agarwal et al., 2014
		Ärnlöv et al., 2013a
		Sharma et al., 2020
		Song et al., 2021
	Not correlated	Reindl et al., 2017
		di Giuseppe et al., 2014
Comorbidities		
Diabetes	Higher prevalence	Lutsey et al., 2014
		Panwar et al., 2015
		Ix et al., 2012
		Wright et al., 2014
		Parker et al., 2010
		Kestenbaum et al., 2014
		Speer et al., 2015
		Wohlfahrt et al., 2015
		Agarwal et al., 2014
		Sharma et al., 2020
		Song et al., 2021
	Not correlated	Masson et al., 2015
		Ärnlöv et al., 2013a
Hypertension	Higher prevalence	Lutsey et al., 2014
		Masson et al., 2015
		Ix et al., 2012
		Kestenbaum et al., 2014
		Wright et al., 2014
		Agarwal et al., 2014
		Sharma et al., 2020
		Patel et al., 2020
	Not corelated	Parker et al., 2010
		Ärnlöv et al., 2013a

#### FGF-23 and Lifestyle: Smoking, Poor Physical Activity, and Inadequate Food Intake

Many authors have found a relationship between high plasma levels of FGF-23 and smoking (Parker et al., 2010; Seiler et al., 2011; Ix et al., 2012; Agarwal et al., 2014; di Giuseppe et al., 2014; Di Giuseppe et al., 2015; Masson et al., 2015; Panwar et al., 2015; Souma et al., 2016; Patel et al., 2020), and only a few authors have failed to find this relationship (Ärnlöv et al., 2013a; Kestenbaum et al., 2014). Furthermore, patients with low levels of physical activity seem to have greater levels of FGF-23 (Ix et al., 2012; Kestenbaum et al., 2014; Masson et al., 2015). Gardinier et al. (2016) also demonstrated a significant increase in FGF-23 levels during exercise, mainly due to the response in osteocytes to the increased expression of PTH. Other studies have not found any changes in FGF-23 levels during exercise, however, even after demonstrating significantly increased Klotho levels and decreased PTH levels (Fakhrpour et al., 2020). A recent study comparing master athletes with age-matched controls showed a significantly better redox balance in athletes, with significantly higher Klotho levels possibly explaining the lower FGF-23 levels in people with higher physical activity (Rosa et al., 2020). It would be interesting to separate physical activity from inadequate food intake, however, as this could also influence FGF-23 levels. In this sense, food preserved with phosphate additives is associated with higher FGF-23 levels (Yuen et al., 2016; Pool et al., 2020). Thus, future studies should further explore the relationship between chronic dietary phosphate intake, FGF-23 levels and their specific association with cardiovascular risk. As smoking, low levels of physical activity and inadequate food intake are cardiovascular risk factors, these factors would enhance the role of FGF-23 in cardiovascular outcomes.

#### FGF-23 and MBD or Kidney Disease

**FIGURE 1:**
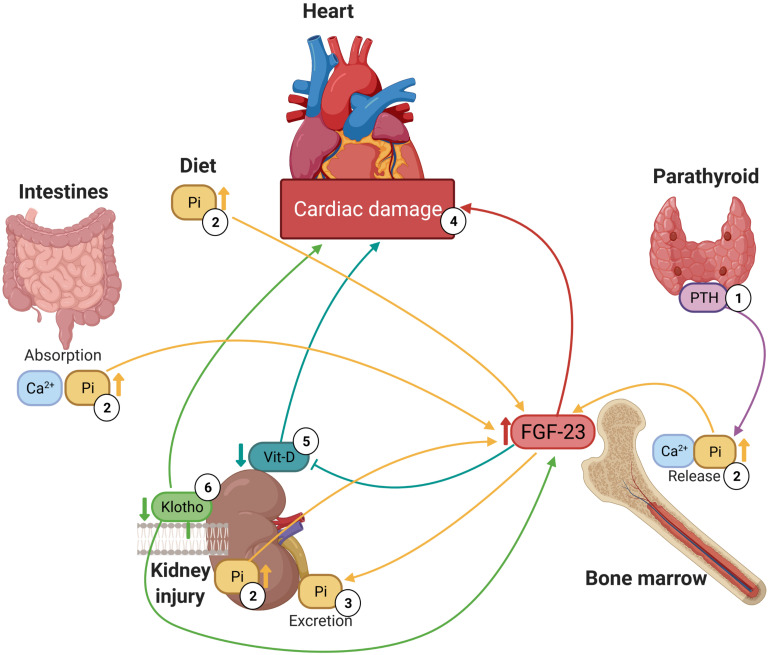
Relationship between high plasma levels of FGF-23, mineral bone metabolism components and cardiac damage. (1) Parathyroid stimulates the release of phosphate and calcium from large calcium stores in the bones into the bloodstream. (2) High plasma levels of phosphate promote FGF-23 synthesis in bone marrow. (3) FGF-23 enhances the excretion of phosphate in kidneys. (4) High circulating FGF-23 levels are associated with several forms as cardiac damage including hypertrophy and HF. (5) FGF-23 reduces circulating 1,25(OH)2D, active form of vitamin D, and deficiency of vitamin D is associated with cardiac damage. (6) Low levels of Klotho are linked to both high plasma levels of FGF-23 and cardiac damage.

Mineral bone disorders are usually the consequence of electrolyte imbalances, i.e., hyperphosphatemia, hyperkalaemia, or hypercalcemia (Navarro-García et al., 2018), which have been associated with high plasma levels of FGF-23. Indeed, elevated serum levels of FGF-23 are related to increased levels of phosphate (Parker et al., 2010; Seiler et al., 2011; Taylor et al., 2011; Shibata et al., 2013; Agarwal et al., 2014; Brandenburg et al., 2014; Kestenbaum et al., 2014; Lutsey et al., 2014; Poelzl et al., 2014; Wright et al., 2014; Panwar et al., 2015; Souma et al., 2016; Reindl et al., 2017), calcium (Ca^2+^) (Parker et al., 2010; Wright et al., 2014; Di Giuseppe et al., 2015; Panwar et al., 2015; Reindl et al., 2017; Patel et al., 2020) and PTH (Taylor et al., 2011; Brandenburg et al., 2014; di Giuseppe et al., 2014; Lutsey et al., 2014; Poelzl et al., 2014; Wright et al., 2014; Di Giuseppe et al., 2015; Souma et al., 2016; Sharma et al., 2020) and low levels of vitamin D (Shibata et al., 2013; Poelzl et al., 2014; Chonchol et al., 2016; Souma et al., 2016). Although it should also be noted that some authors did not find a relationship with Ca^2+^ (Agarwal et al., 2014), PTH (Shibata et al., 2013) or vitamin D (Taylor et al., 2011; Kestenbaum et al., 2014; Masson et al., 2015). It is well known that there is a complex network between FGF-23, PTH, vitamin D, calcium, phosphates and Klotho levels. This physiological network consists of a feedback mechanism through which disturbances in the level of one of these substances leads to changes in the levels of all the others (Navarro-García et al., 2018). Therefore, increased levels of PTH or vitamin D stimulate the synthesis of FGF-23 in the long bones and reduced renal Klotho expression increases serum phosphate load, which also induces FGF-23 synthesis. These mineral disturbances are classic complications in CKD, but in the last few decades, it has been shown that they also have a huge impact on cardiac tissue (Figure 1) because they have direct and indirect cardiotoxic effects, such as the development of LVH, HF or arrhythmias like AF (Navarro-García et al., 2018).

In relation to kidney disease, high plasma levels of FGF-23 have also been associated with impaired kidney function as represented by an estimated glomerular filtration rate (eGFR) < 60 mL/min/1.73 m^2^ (Parker et al., 2010; Seiler et al., 2011; Ix et al., 2012; Shibata et al., 2013; Ärnlöv et al., 2013a, b; Agarwal et al., 2014; Brandenburg et al., 2014; Kestenbaum et al., 2014; Lutsey et al., 2014; Poelzl et al., 2014; Wright et al., 2014; Koller et al., 2015; Panwar et al., 2015; Speer et al., 2015; Wohlfahrt et al., 2015; Souma et al., 2016; Ter Maaten et al., 2018; von Jeinsen et al., 2019; Patel et al., 2020; Sharma et al., 2020) and high levels of creatinine (Taylor et al., 2011; Ix et al., 2012; Shibata et al., 2013; Ärnlöv et al., 2013a; Agarwal et al., 2014; Kestenbaum et al., 2014; Wright et al., 2014; Koller et al., 2015; Masson et al., 2015; Panwar et al., 2015; Speer et al., 2015; Souma et al., 2016; Sharma et al., 2020; Song et al., 2021). di Giuseppe et al. (2014); Andersen et al. (2016), and Reindl et al. (2017) did not find evidence of this association; however, the number of subjects they studied was limited. CKD is characterised by excessive plasma levels of phosphate; therefore, increased synthesis of FGF-23 is a compensatory mechanism that preserves phosphate homeostasis by increasing urinary excretion. FGF-23 decreases the activity of the sodium-phosphate cotransporter in the renal proximal tubule and reduces dietary absorption by down-regulating the expression of the enzyme responsible for synthesising calcitriol, 1α-hydroxylase (Gutiérrez et al., 2008; Wolf, 2012). Therefore, high plasma levels of FGF-23 are a good biomarker for impaired renal function.

#### Other Comorbidities Associated With High Plasma Levels of FGF-23.

Two of the major comorbidities that contribute to the development of cardiovascular disease are diabetes and hypertension, both of which now have a high prevalence (Ruilope, 2012; Strain and Paldanius, 2018). Recent studies have associated elevated plasma levels of FGF-23 with a higher prevalence of diabetes mellitus (Parker et al., 2010; Ix et al., 2012; Agarwal et al., 2014; Kestenbaum et al., 2014; Lutsey et al., 2014; Wright et al., 2014; Panwar et al., 2015; Speer et al., 2015; Wohlfahrt et al., 2015; Sharma et al., 2020; Song et al., 2021) and hypertension (Ix et al., 2012; Agarwal et al., 2014; Kestenbaum et al., 2014; Lutsey et al., 2014; Wright et al., 2014; Masson et al., 2015; Patel et al., 2020; Sharma et al., 2020). Some studies have demonstrated a reduction in soluble Klotho levels in patients with impaired fasting glucose (Zhang and Liu, 2018), together with increased levels of FGF-23 (Zaheer et al., 2017) in diabetic patients compared to healthy volunteers, even with preserved kidney function. In the field of hypertension, Liu et al. and other studies have found an association between FGF-23 and arterial stiffness after renal transplantation when renal function is restored (Feng et al., 2020; Liu et al., 2020). These comorbidities contribute to an increased risk of mortality and adverse outcomes and worsen the quality of life of patients and might be explained by the cardiovascular effects of FGF-23. Some studies did not support this association (Parker et al., 2010; Ärnlöv et al., 2013a; Masson et al., 2015), however. Further studies are needed to clarify the independence of hypertension and diabetes in elevating systemic levels of FGF-23.

In conclusion, high plasma levels of FGF-23 are associated with certain demographics, such as age and sex; with cardiovascular diseases like LVH, HF and AF; with clinical disturbances such as low eGFR, high phosphate and NT-pro-BNP; and with comorbidities like diabetes and hypertension. All these studies point to FGF-23 as an important and independent biomarker involved in several pathologic processes, including cardiovascular disease. This idea must be interpreted with caution, however, as contradictions remain in the literature and a clear causal relationship is yet to be established, at least in a clinical setting. Hence, there is a need to experimentally address the relationship between high plasma levels of FGF-23 and cardiac disturbances to shed light on the origin of FGF-23 as a cause or consequence of cardiac damage and identify the specific FGF-23 downstream molecular pathways involved.

## FGF-23 Induces Cardiac Damage: Experimental *in vitro* and *in vivo* Approaches

As already mentioned, over the last decade, many studies have shown a significant and independent relationship between high plasma levels of FGF-23 and detrimental cardiac outcomes in a clinical setting (Table 2). In this cardiovascular setting, an urgent roadmap for further research has emerged, and experiments aimed at unravelling the role of FGF-23 in cardiac pathology are needed. To date, research studies have demonstrated that FGF-23 might induce cardiac dysfunction, hypertrophy and fibrosis in the heart.

### FGF-23 Causes Cardiac Dysfunction

Correct heart function depends directly on the functional cells of the heart, that is, on the precise and coordinated contraction and relaxation cycle of cardiomyocytes (Fozzard, 1991). During contraction, intracellular Ca^2+^ levels of cardiomyocytes increase due to changes in the cardiomyocyte membrane action potential (AP) (Bers, 2002). AP is the reversible change in membrane potential as a consequence of the opening of different ion channels and leads to cardiomyocyte contraction in a process called excitation–contraction (EC) coupling, which represents the translation of electrical stimulation into mechanical work (Bers, 2002). Ca^2+^ is a key player in cardiac EC coupling (Fabiato and Fabiato, 1975) as a rise in free cytosolic Ca^2+^ is essential for the contraction of the cardiomyocytes (Bers, 2002). L-type calcium channels at the sarcolemma open due to the depolarisation of the AP, allowing the first Ca^2+^ influx into the cytoplasm (Fabiato and Fabiato, 1975). This Ca^2+^ influx activates ryanodine receptors (RyRs), triggering a greater amount of Ca^2+^ release from the sarcoplasmic reticulum (SR) to the cytoplasm (Bers, 2006). The intracellular Ca^2+^ concentration increases and Ca^2+^ binds to troponin C at the myofilaments, starting the cardiomyocyte contraction (Bers, 2006).

To allow physiological relaxation, the Ca^2+^ levels should decrease in the same amount as the previous increase (Eisner et al., 2017). This reduction of cytosolic Ca^2+^ mostly takes place via two different processes: 92% of cytosolic Ca^2+^ is pumped back to the SR by the action of the SR–Ca^2+^–adenosine triphosphatase 2a (SERCA) and, to a lesser extent (7%), Ca^2+^ is extruded from the cytoplasm to the extracellular medium by the Na^+^–Ca^2+^ exchanger (Bers, 2006). This Ca^2+^ handling in the cardiomyocytes is a complex physiological process perfectly regulated by different proteins, and it is well known that calmodulin kinase II (CaMKII) and cAMP-dependent protein kinase (PKA) regulate the open probability of RyRs through several phosphorylations at different sites, increasing their activity (Bovo et al., 2017). In addition, CaMKII and PKA can also phosphorylate phospholamban (PLB), a SR membrane protein that regulates SERCA activity. PLB binds to SERCA and inhibits its activity in situations of dephosphorylation (Kranias and Hajjar, 2012).

**FIGURE 2:**
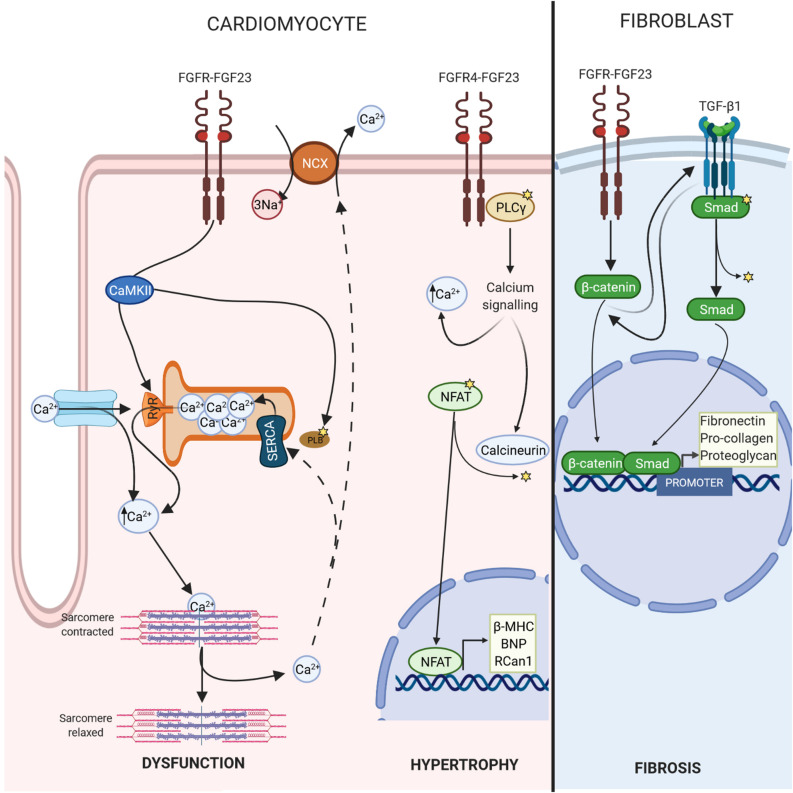
FGF-23-FGFR signalling. FGF-23 binds to the extracellular domain of its receptor, causing autophosphorylation. In cardiomyocytes, this change leads to phosphorylate calcium regulatory proteins, such as CAMKII, which promotes Ca^2+^ mishandling and cardiac dysfunction. In addition, FGF-23/FGFR4 triggers calcium cascade and the activation of calcineurin, which dephosphorylate NFAT inducing transcription of pro-hypertrophic genes such as β*-MHC*, *BNP*, or *Rcan1*. In fibroblasts, FGF-23 promotes fibrosis through activation of β-catenin.

In this context, it has been shown that FGF-23 is involved in this Ca^2+^ handling by increasing phosphorylation of regulator proteins such as CaMKII (Kao et al., 2014; Navarro-García et al., 2019), which could drive cardiomyocytes to develop a cellular phenotype related to contractile dysfunction and predisposition to arrhythmias (Curran et al., 2010) (Figure 2). Recent works have demonstrated that FGF-23 might induce pro-arrhythmogenic Ca^2+^ events by itself in isolated adult rat ventricular cardiomyocytes due to alterations in Ca^2+^ handling (Navarro-García et al., 2019). Direct acute perfusion of 100 ng/mL FGF-23 reduced systolic Ca^2+^ release and increased the spontaneous diastolic Ca^2+^ leak, increasing the incidence of arrhythmogenic Ca^2+^ events (Navarro-García et al., 2019). This phenotype is frequently observed in failing cardiomyocytes (Ruiz-Hurtado et al., 2015; Alvarado et al., 2017; Chen et al., 2020), which might explain the relationship between FGF-23 levels and HF incidence described previously in this review. The events observed in cells are strongly influenced by Ca^2+^ mishandling and could be induced by RyRs hyperactivity and a reduction in SERCA activity, both of which are responsible for reduced SR Ca^2+^ load (Navarro-García et al., 2019). These alterations would lead to impaired cardiomyocyte contraction, represented by less cell shortening (Navarro-García et al., 2019).

Similar results related to cardiac dysfunction and predisposition to ventricular arrhythmia were found in nephrectomised mice and homozygous Klotho-hypomorphic (*kl/kl*) mice, both of which are experimental animal models that have middle (∼300 pg/mL) or very high (∼400.000 pg/mL) circulating FGF-23 levels (Navarro-García et al., 2020). Interestingly, when these mice are treated with recombinant Klotho, cardiac dysfunction and pro-arrhythmogenic Ca^2+^ release events are prevented (Navarro-García et al., 2020). This highlights the possible protection of Klotho against the deleterious effects of FGF-23. Moreover, other studies reported that the cellular HL-1 line of atrial murine cardiomyocytes had a greater L-type Ca^2+^ current and greater Ca^2+^ transients and SR Ca^2+^ content after maintained FGF-23 (25 ng/mL) exposure during 24 h of treatment compared to control cells (Kao et al., 2014). This higher SR Ca^2+^ content may be a consequence of increased SERCA activity due to phosphorylation of PLB at the CaMKII-specific site (Thr^17^) and not due to an intrinsic increase in the protein expression of SERCA or PLB (Kao et al., 2014). FGF-23 also dysregulated Ca^2+^ homeostasis, triggering delayed after depolarizations that have been related to the initiation of ventricular arrhythmias (Kao et al., 2014).

It is important to note that although both the cellular studies described above showed apparently different effects of FGF-23, the first showed the acute effect of FGF-23 on ventricular cardiomyocytes from a rat, while the second was carried out on an atrial cell line exposed to FGF-23 for longer periods. In both cases, the conclusion was that FGF-23 led to a greater predisposition to arrhythmias. Another effect of FGF-23 was described using mice ventricular muscle strips and showed that FGF-23 (9 ng/mL) was able to induce a significant increase in the peak isometric tension, slope and area of contractile waveforms (Touchberry et al., 2013). Therefore, in addition to the effect of FGF-23 on predisposition to cellular arrhythmia, it also could promote important alterations in cardiac function that regulate the contraction and relaxation process. It is well known that the main action of FGF-23, its phosphaturic action in the kidney, takes place via FGFR-binding; however, the FGFR isoform responsible for this effect of FGF-23 on cardiac function is yet to be identified, although some research has pointed to FGFR1 (Touchberry et al., 2013; Schumacher et al., 2019) or FGFR4 (Faul et al., 2011; Faul, 2017; Grabner et al., 2017).

Electrocardiogram (ECG) and echocardiography are two non-invasive methods used to estimate *in vivo* cardiac function. Ca^2+^ mishandling causes important changes in EC coupling, and this could also lead to pro-arrhythmogenic activity of cardiomyocytes, which is associated with abnormal heart rhythms. Indeed, a single-dose FGF-23 injection (40 μg/kg) in healthy mice was able to induce a significant increase (60%) in the occurrence of premature ventricular contractions recorded by ECG (Navarro-García et al., 2019). In addition, echocardiography allows the LV dimensions during systole and diastole, fractional shortening and ejection fraction to be measured. In general, mice with high circulating levels of FGF-23 had worse cardiac function than control mice, showing a lower percentage of fractional shortening, larger LV internal diameter in diastole, less cardiac output or higher LV diastolic pressure (Faul et al., 2011; Touchberry et al., 2013; Andrukhova et al., 2015; Hao et al., 2016; Han et al., 2020). Hence, FGF-23 could be an important factor causing changes in cardiac function and heart rhythm that can lead to the onset and worsening of cardiac disease.

### FGF-23 Induces LVH

Ca^2+^ abnormalities, usually those related to Ca^2+^ overload, precede cardiac dysfunction, while LVH develops later (Li et al., 2019). LVH, which is present in 15–20% of the general population, is one of the main risk factors for the development of a variety of adverse cardiac events and is related to pathological cardiac remodelling (Weber, 1991). It has already been demonstrated that FGF-23 promotes cardiac hypertrophy directly by acting on FGFR4, which subsequently triggers the phospholipase (PLC)γ/calcineurin/nuclear factor of activated T-cells (NFAT) signalling pathway (Faul et al., 2011; Grabner et al., 2015; Leifheit-Nestler et al., 2018b; Han et al., 2020). FGF-23 binds to FGFR4, causing its auto-phosphorylation, which then induces PLCγ activation through the binding of PLCγ to a specific phosphorylated tyrosine residue within the FGFR4 cytoplasmic sequence (Vainikka et al., 1994). Activated PLCγ increases cytoplasmic Ca^2+^ levels and activates calcineurin, the main regulator of intracellular Ca^2+^ (Eswarakumar et al., 2005), with the subsequent dephosphorylation of NFAT (Molkentin, 2004). Dephosphorylated NFAT translocates to the nucleus, where it promotes the transcription of pro-hypertrophic genes, including regulator of calcineurin 1 (*Rcan1*), brain natriuretic peptide (*BNP*), atrial natriuretic peptide (*ANP*) and β-myosin heavy chain (β*-MHC*) (Han et al., 2020) (Figure 2).

The hypertrophic effect of FGF-23 has been proven in *in vitro* and *in vivo* experiments. HL-1 atrial cells incubated with recombinant FGF-23 (rFGF-23) exhibited a higher cross-sectional surface area and an increase in pro-hypertrophic genes (Touchberry et al., 2013). Similarly, murine models with high levels of circulating FGF-23 showed greater mRNA levels of pro-hypertrophic genes, greater heart weight/body weight or heart weight/tibial length ratios and greater LV thickness (Faul et al., 2011; Andrukhova et al., 2015; Hao et al., 2016; Slavic et al., 2017; Leifheit-Nestler et al., 2018b; Han et al., 2020). Faul et al. (2011) published a large study where they demonstrated the hypertrophic effect of FGF-23. Cell surface area and expression of pro-hypertrophic genes in isolated neonatal rat ventricular myocytes (NRVM) increased after 48 h of treatment with FGF-23 (10, 25, and 100 ng/mL), while direct administration of rFGF-23 into the LV myocardium in mice caused cardiac hypertrophy after 14 days of treatment, even without an increase in circulating FGF-23 levels or other changes in mineral metabolism (Faul et al., 2011). Mice injected with FGF-23 showed greater heart weight/tibial length ratios and LV wall thickness in contrast to vehicle mice (Faul et al., 2011). This cardiac remodelling was also observed in mice after rFGF-23 (40 μg/kg) intravenous injections twice daily for 5 days, after which the immunohistochemistry and RT-PCR of pro-hypertrophic genes in the heart showed the presence of LVH development (Faul et al., 2011). Han et al. (2020) reported similar results in mice after intraperitoneal administration of rFGF-23 at 75 ng/g twice daily for 5 days. Leifheit-Nestler et al. (2018b) studied two different mouse strains: *kl/kl* mice and an experimental model for human X-linked hypophosphatemia with a mutation that inactivates the endopeptidases of the X chromosome (Hyp) mice. Both models showed high endogenous FGF-23 synthesis and elevated circulating FGF-23 levels compared to wild-type (WT) controls. *kl/kl* mice exhibited adverse cardiac remodelling, their heart weight/body weight ratios and cross-sectional areas of myocytes were higher than those of WT mice and the cardiac mRNA of their pro-hypertrophic genes was also increased. In the same way, experimental models of cardiac damage, such as post MI, ischemia reperfusion (I/R) and transversal aortic constriction, have been used to demonstrate that LVH is related to a profound increase in circulating levels of FGF-23, higher cardiac mRNA and protein expression of FGF-23 (Andrukhova et al., 2015; Hao et al., 2016; Slavic et al., 2017). All these data point to FGF-23 as a direct cause of LVH.

Furthermore, several studies have provided evidence that FGF-23 activates the PLCγ/calcineurin/NFAT signalling pathway. *In vivo*, healthy mice treated with rFGF-23 showed upregulated PLCγ protein levels compared to vehicle-treated mice (Han et al., 2020). In addition, *in vitro* treatment of rFGF-23 in NRVM activates PLCγ, and incubation with the PLCγ inhibitor U73122 blocks the hypertrophic effect of FGF-23 (Faul et al., 2011). Other evidence has been reported by Faul et al. (2011) and Grabner et al. (2015), who analysed NFAT activity after incubation with rFGF-23 in C2C12 myoblast (an immortalised mouse myoblast cell line) and in NRVM, respectively, and found that cells treated with FGF-23 showed a rise in NFAT activity compared to vehicle-treated cells. Also, Grabner et al. (2015) showed that this effect of FGF-23 was blocked by the calcineurin inhibitor cyclosporine A. In a study by Leifheit-Nestler et al. (2018b), qRT-PCR analysis revealed that cardiac FGFR4 mRNA expression was upregulated in both *kl/kl* and Hyp mice compared to their respective WT controls, but especially in *kl/kl* mice. In contrast, calcineurin protein levels were increased and NFAT phosphorylation was reduced only in *kl/kl* mice, therefore, the pro-hypertrophic genes were upregulated in *kl/kl* mice but not Hyp mice. There are two likely reasons for the differences observed between mouse strains. First, the circulating levels of FGF-23 are much higher in *kl/kl* mice. Second, the differences in Ca^2+^ and phosphate levels: *kl/kl* mice present with hyperphosphatemia and hypercalcemia, while Hyp mice have hypophosphatemia and normal levels of Ca^2+^. Interestingly, this hypertrophic effect is also influenced by the absence of Klotho, as *kl/kl mice*, which is the experimental model with the highest systemic FGF-23 levels, show a greater degree of hypertrophy in a Klotho-deficient context. It is also important to mention that soluble Klotho could act as a circulating lure for FGF-23, impeding its binding to cardiac FGFRs; act as a coreceptor in cardiomyocytes, causing a switch in or even the blockade of the FGF-23 signalling pathway; or interact with an unknown receptor activating the cardiac protective signalling pathway. Thus, Klotho could block or reduce the hypertrophic effect of FGF-23 on the heart, as it does with the deleterious effect of FGF-23 on cardiac function (Navarro-García et al., 2019, 2020). Indeed, several authors have shown the cardioprotective action of recombinant supplementation with Klotho in several experimental models of direct or indirect cardiac damage (Xie et al., 2012; Hu et al., 2017; Liu et al., 2019; Navarro-García et al., 2019, 2020; Wright et al., 2019; Han et al., 2020).

FGFR4 expression is upregulated when FGF-23 levels are elevated, therefore, FGF-23 is considered to be an inducer of cardiac hypertrophy through FGFR4 (Grabner et al., 2015). Immunoprecipitated PLCγ-bound FGFR4 was elevated in isolated NRVM treated for 30 min with FGF-23 at 25 ng/mL compared to control cells and cells co-treated with FGF-23 plus anti-FGFR4 antibodies (Grabner et al., 2015). Although a high phosphate (2%) diet is known to induce LVH and FGF-23 synthesis (Hu et al., 2015), FGFR4 knockout (KO) mice did not show LVH after 12 weeks of a high phosphate diet. In this way, blocking FGFR4 could be potentially useful for avoiding deleterious FGF-23 cardiac actions in a clinical setting (Grabner et al., 2015). Grabner et al. (2017) attributed a substantial role in LVH pathogenesis to FGFR4 and claimed that FGF-23/FGFR4-mediated LVH could be reversible, at least in rodents. To validate this hypothesis, they carried out three types of experiments. The first found that the hypertrophic effect was reversible after incubation of NRVM with FGF-23 for 24 h and then with anti-FGFR4 antibodies. In addition, pre-treatment with anti-FGFR4 antibodies prevented FGF-23-induced hypertrophy. In the second experiment, LVH induced by 5/6 nephrectomy in rats, a model that presents high FGF-23 levels, did not progress in animals treated with anti-FGFR4 antibodies for 4 weeks after surgery compared with untreated nephrectomised rats. In the third experiment, 18-month-old FGFR4 KO mice were protected from LVH (despite having high plasma levels of FGF-23) compared with WT mice, which, in normal conditions, at this age develop LVH (Grabner et al., 2017). Finally, a recent study using inducible FGFR4 KO mice in which FGFR4 was depleted only in the heart by tamoxifen treatment, found that they did not develop LVH after intraperitoneal injections of rFGF-23 at 75 ng/g twice daily for 5 days, unlike WT mice given the same treatment (Han et al., 2020). All these experimental studies show that FGF-23 activates cardiac PLCγ/calcineurin/NFAT signalling and development of LVH through FGFR4 (Figure 2).

### FGF-23 Induces Cardiac Fibrosis

Fibrosis is a response aimed at protecting an organ from an injurious event; however, it leads to massive deposition of the extracellular matrix (ECM), disrupting the normal tissue architecture and inducing destruction of the parenchyma (Franklin, 1997). Tissue fibrosis is responsible for increased organ size; therefore, an increase in the heart’s mass can be due to cellular hypertrophy or cardiac fibrosis or both. The cellular hypertrophic effect of FGF-23 has already been established (see section 3.2); however, the role of circulating and/or cardiac FGF-23 in the progression of cardiac fibrosis is not yet clear. Hao et al. (2016) suggested that FGF-23 promotes myocardial fibrosis through the activation of β-catenin, which is a profibrotic factor that cross-talks with transforming growth factor-β1 (TGF-β1) signalling, also an important fibrogenic agent. TGF-β1 receptors/Smad complexes stimulate chemotaxis in fibroblasts and increase the expression of collagen, fibronectin, and proteoglycans, the main components of the ECM (Leifheit-Nestler and Haffner, 2018). TGF-β1 signalling can induce the expression of β-catenin pathway members and *vice versa* (Guo et al., 2012). When β-catenin translocases into the nucleus, Smads and β-catenin form a complex at the promoter to coregulate specific gene expression and stimulate the transcription of fibrosis-related genes (Guo et al., 2012) (Figure 2). In adult myocardial fibroblasts, rFGF-23 induces proliferation in a dose-dependent manner (15, 25, and 50 ng/mL) through overexpression of profibrotic genes, such as β-catenin and procollagen I and II (Hao et al., 2016). Furthermore, FGF-23 increases the proliferation of neonatal rat cardiofibroblasts and expression of TGF-β1, TGF-β1 receptor/Smad complexes, connective tissue growth factor and collagen I (Leifheit-Nestler et al., 2018a; Böckmann et al., 2019). It has been shown that FGF-23 also intensifies the effect of TGF-B1 via FGFR1 promoting fibrosis, whereas cotreatment with an inhibitor of FGFR1 avoids this fibrosis (Kuga et al., 2020).

Böckmann et al. (2019) also hypothesised that FGF-23 might activate the local renin–angiotensin–aldosterone system, promoting fibrosis through increased cardiac mRNA expression levels of angiotensinogen, angiotensin-converting enzyme and angiotensin II receptor type 1. This hypothesis has already been demonstrated via the cotreatment of neonatal rat cardiofibroblasts with antihypertensive drugs like spironolactone, which prevented fibrotic development (Böckmann et al., 2019). Therefore, further studies are needed to unravel the different pathways through which FGF-23 induces cardiac fibrosis.

FGF-23-induced cardiac fibrosis has also been observed using *in vivo* experimental approaches. Leifheit-Nestler et al. (2018b) showed that *kl/kl* mice developed myocardial fibrosis due to a significant rise in the expression of collagen I and TGF-β1. Furthermore, Hu et al. (2015) showed that TGF-β1 is responsible for activating the extracellular signal-regulated kinase 1/2 pathway in *kl/kl* mice, which leads to cardiac hypertrophy and fibrosis. It has been demonstrated that direct damage to the myocardium raises cardiac levels of FGF-23 (Schumacher et al., 2019). In an MI mice model, FGF-23 and FGFR1 increased early in the myocardium (Schumacher et al., 2019). This cardiac FGF-23 could result from an increased uptake from the circulation or may be synthesised intrinsically in the heart (Schumacher et al., 2019). The main sources of FGF-23 in the heart are local fibroblasts, which increase fibroblast migration and the expression of fibronectin and collagen I (Schumacher et al., 2019). Hao et al. (2016) used adeno-associated virus-carrying FGF-23 injections in mice followed by MI or I/R. The treated mice showed significantly increased cardiac fibrosis compared to negative controls, and this effect was prevented by the inhibition of β-catenin (Hao et al., 2016). FGF-23 might have a positive role in healing after an MI by stimulating the fibroblasts to fill the empty area and produce ECM components (Schumacher et al., 2019); however, it induces pathologic fibrosis in the heart after long exposures and can also influence cardiac hypertrophy development.

## Strategies to Avoid Deleterious FGF-23 Effects on the Heart

Various therapeutic approaches are currently being considered to avoid the harmful effects of FGF-23. The key aspects are listed here as follows: FGF-23 antibodies, blockers of FGFRs and phosphate binders. In addition, Klotho as a potential therapy has been included based on the relevant cardio-protection showed by the exogenous recombinant Klotho supplementation examined in several experimental studies.

### FGF-23 Antibodies

FGF-23 antibodies could be considered as the first therapeutic option; however, it is important to point out that FGF-23 is a pleiotropic factor that regulates many physiological processes, such as phosphate excretion. These processes would also be blocked with the use of these antibodies. For example, chronic treatment with FGF-23 antibodies for 6 weeks in an experimental CKD rat model improved secondary hyperparathyroidism by decreasing PTH and increasing vitamin D, but phosphate levels, aortic calcification and even mortality were significantly increased (Shalhoub et al., 2012). Therefore, this therapeutic option can be dismissed as the negative impact of the side effects might be greater than the clinical benefits as in situations of renal dysfunction. Currently, these antibodies have only been validated for the treatment of X-linked hypophosphatemic rickets caused by high plasma levels of FGF-23 (Carpenter et al., 2014). There have been several studies in humans (Carpenter et al., 2014; Imel et al., 2015) and Hyp mice (Yamazaki et al., 2008; Aono et al., 2009, 2011) that support the efficacy of FGF-23 antibodies in ameliorating biochemical, morphological, histological and clinical abnormalities associated with X-linked hypophosphatemia, but the safety of this treatment is still under investigation (Fukumoto, 2016) (reported at www.clinicaltrials.gov: NCT02163577 and NCT02312687).

### FGFRs Blockers

FGF-23 exerts its action by binding to FGFRs 1–4, and several studies have proposed FGFR4 as the main FGFR isoform that mediates the effect of FGF-23 on the heart (Grabner et al., 2015, 2017; Leifheit-Nestler et al., 2016). Therefore, a useful therapeutic option currently being studied to impede FGF-23 effects on the heart consists of the specific blockage of FGFR4 (Grabner et al., 2017). This receptor has been related to the deleterious cardiovascular effect mediated by FGF-23, such as LVH inducing the overexpression of cardiac remodelling genes, including *BNP* and β*-MHC* (Leifheit-Nestler et al., 2018b). Moreover, the phosphaturic action of FGF-23 is mediated by FGFR1 in the kidneys. Therefore, blocking FGFR4 could prevent the adverse effects of FGF-23 on the heart without interfering in the other physiological functions of FGF-23, such as increased phosphate excretion in situations where the renal function has declined (Taylor et al., 2019). In support of this, Grabner et al. (2017) showed *in vitro* in NRVM that the hypertrophic effect of FGF-23 can be prevented by pre-treating these cells with anti-FGFR4. Furthermore, in the same study, LVH did not progress in 5/6 nephrectomised rats treated with anti-FGFR4 antibodies for 4 weeks after surgery compared with the control group (in which a further 28% increase in LVM was observed) (Grabner et al., 2017). These experimental results make FGFR4 an interesting therapeutic target, the safety of which has already been proven in clinical trials in cardiovascular diseases (reported at www.clinicaltrials.gov: NCT02476019). In addition, FGFR4 blockade is currently used in oncology trials as neoadjuvant chemotherapy to improve patients’ response, since it has a beneficial anti-angiogenic effect (Xin et al., 2018; Gluz et al., 2020).

### Phosphate Binders

High plasma levels of FGF-23 are correlated with high levels of phosphate, which are also considered to be a cause of cardiovascular adverse events (Zoccali et al., 2013). In this way, decreasing phosphate levels could improve the prognosis of patients with cardiovascular disease who have high plasma levels of FGF-23. There are three common types of phosphorus binders: Ca^2+^-containing binders, aluminium-containing binders and non-Ca^2+^ or Ca^2+^-free phosphate binders (Chan et al., 2017). These drugs are commonly used in the CKD population in combination with a dietary phosphate restriction (Ketteler and Biggar, 2013). Sharon et al. showed that patients with CKD and hyperparathyroidism treated with cinacalcet for 20 weeks had a 30% decrease in their levels of FGF-23 compared with the placebo group, and this was associated with a lower risk of cardiovascular events and mortality (Moe et al., 2015). In another study, Gonzalez-Parra et al. (2011) showed that treatment with the phosphate binder lanthanum carbonate in stage 3 CKD patients was associated with a decrease in serum FGF-23 levels and a decrease in the risk of adverse cardiovascular events. Nonetheless, the effect of these treatments is modest, and the levels of FGF-23 remain above the normal range despite the reported decreases (Zoccali et al., 2013).

### Klotho as a Potential Therapeutic Option

Recent experimental research has pointed to Klotho as a potentially useful treatment for reducing or avoiding the harmful effects of FGF-23 on the heart. Indeed, Han et al. (2020) showed that the FGF-23/FGFR4 signalling is attenuated by intraperitoneal administration of soluble Klotho in mice, preventing FGF-23-induced LVH. Moreover, Hu et al. (2015) and Navarro-García et al. (2020) used a *kl/kl* mice to show that Klotho deficiency is a novel intermediate mediator of pathologic cardiac remodelling and dysfunction induced by high plasma levels of FGF-23. There are three hypothetical actions by which Klotho could prevent the effects of FGF-23 on the heart: by being a soluble lure for FGF-23 and impeding its binding to cardiac FGFR4; by acting as a coreceptor in cardiomyocytes, causing a switch in or even inhibition of the FGF-23 signalling pathway; or by interacting with an unknown receptor and activating the cardiac protective signalling pathway. To date, Klotho therapy has been studied only at the experimental level. Further research is needed to determine the ability of Klotho therapy to prevent the detrimental effects of FGF-23 in patients while maintaining the physiological phosphaturic FGF-23 action.

In summary, more experimental research and clinical data are required to identify which is the best option for controlling the deleterious effects of FGF-23, especially on the heart, while maintaining its physiological phosphaturic action. This option could involve blocking the FGF-23/FGFR4 axis or it could involve the promising therapies that use recombinant Klotho.

## Concluding Remarks and Future Perspectives

In conclusion, FGF-23 has a direct association with the development and progression of several events related to high cardiovascular risk, such as LVH, HF and arrhythmias, and its value as a cardiovascular biomarker in humans has been clinically demonstrated. Furthermore, a variety of *in vitro* and *in vivo* experimental approaches have indicated that FGF-23/FGFR axis signalling, especially that of the FGF-23/FGFR4 axis, provides an important base for the development of cardiac dysfunction through Ca^2+^ mishandling and cardiac hypertrophy and cardiac fibrosis through increased expression of target genes and proteins. Therefore, this axis represents an excellent therapeutic target for interrupting the harmful cardiac effects of FGF-23. Indeed, diverse neutralising anti-FGF-23 or anti-FGFR blocking antibodies are being tested in human clinical trials to assess their utility and safety. Whether FGF-23/FGFR signalling *per se* can initiate changes in cardiac function and structure or whether it only modulates cardiac remodelling in concert with other cardio-toxic factors remains unclear, however. Further research is needed to define whether FGF-23 effects are only deleterious or are dose- and/or time-dependent profitable effects, whether cardiomyocytes may themselves produce FGF-23, and the role of FGF-23 in myocardial damage. Therefore, it is essential to identify all the FGF-23-dependent pathways and their mediators that may have potential diagnostic, prognostic and therapeutic significance to cardiovascular diseases.

## Author Contributions

SV-S, JP, JAN-G, and GR-H wrote the manuscript with the contribution of LG-L, ER-S, and LR. All authors have read and agreed to the published version of the manuscript.

## Conflict of Interest

The authors declare that the research was conducted in the absence of any commercial or financial relationships that could be construed as a potential conflict of interest.
